# Molecular Elucidation of Riboflavin Production and Regulation in Candida albicans, toward a Novel Antifungal Drug Target

**DOI:** 10.1128/mSphere.00714-20

**Published:** 2020-08-05

**Authors:** Liesbeth Demuyser, Ilse Palmans, Paul Vandecruys, Patrick Van Dijck

**Affiliations:** a VIB-KU Leuven Center for Microbiology, Flanders, Belgium; b Laboratory of Molecular Cell Biology, Institute of Botany and Microbiology, KU Leuven, Leuven-Heverlee, Belgium; University of Georgia

**Keywords:** *Candida albicans*, antifungal agents, drug targets, riboflavin, vitamin B_2_

## Abstract

Candida albicans is a major cause of fungal infections, both superficial and invasive. The economic costs as well as consequences for patient welfare are substantial. Only a few treatment options are available due to the high resemblance between fungal targets and host molecules, as both are eukaryotes. Riboflavin is a yellow pigment, also termed vitamin B_2_. Unlike animals, fungi can synthesize this essential component themselves, thereby leading us to appreciate that targeting riboflavin production is a promising novel strategy against fungal infections. Here, we report that the GTP cyclohydrolase encoded by C. albicans *RIB1* (*CaRIB1*) is essential and rate-limiting for production of riboflavin in the fungal pathogen. We confirm the high potential of *Ca*Rib1 as an antifungal drug target, as its deletion completely impairs *in vivo* infectibility by C. albicans in model systems. Furthermore, the stimulating effect of iron deprivation and PKA activation on riboflavin production seems to involve *Ca*Rib1 and the upstream transcription factor *Ca*Sef1. Gathering insights in the synthesis mechanism of riboflavin in pathogenic fungi, like C. albicans, will allow us to design a novel strategy and specifically target this process to combat fungal infections.

**IMPORTANCE** Candida albicans is an important fungal pathogen causing common superficial infections as well as invasive diseases with an extremely high morbidity and mortality. Antifungal therapies are limited in efficiency and availability. In this research, we describe the regulation of riboflavin production in C. albicans. Since riboflavin biosynthesis is essential to this organism, we can appreciate that targeting it would be a promising new strategy to combat these fungal infections. We provide evidence that one particular enzyme in the production process, *Ca*Rib1, would be most promising as an antifungal drug target, as it plays a central role in regulation and proves to be essential in a mouse model of systemic infection.

## Abstract (precis)

Candida albicans is an important fungal pathogen causing common superficial infections as well as invasive diseases with an extremely high morbidity and mortality. Antifungal therapies are limited in efficiency and availability. In this research, we describe the regulation of riboflavin production in C. albicans. Since riboflavin biosynthesis is essential to this organism, we can appreciate that targeting it would be a promising new strategy to combat these fungal infections. We provide evidence that one particular enzyme in the production process, *Ca*Rib1, would be most promising as an antifungal drug target, as it plays a central role in regulation and proves to be essential in a mouse model of systemic infection.

## INTRODUCTION

Candida albicans is an opportunistic commensal, becoming pathogenic when the host immune system is compromised. About 70% of women worldwide suffer from vaginal candidiasis at least once in their life. Five to 10% suffer from recurrent vaginal infections, greatly diminishing patient welfare (1, 2). In cases of severe immune deprivation, such as after organ transplantation, the fungus can penetrate host tissue to reach the bloodstream and, from there, reach vital organs. Systemic infections cause death in 40 to 60% of affected patients (3). The limited availability of antifungal therapies and the increased onset of resistance against used drugs hamper efficient eradication of infections and treatment of patients. Despite many attempts made by the scientific community, very few antifungal drugs with a novel mode of action have reached the clinical phase of drug testing over recent years, indicating a need for alternative drug targets (4). A main hurdle when exploring metabolic pathways as potential drug targets is the high level of conservation between the fungal actors and the human ones, as both are eukaryotes. Drugs targeting conserved processes generally exhibit significant side effects (5).

A process that is gaining interest as a potential target for novel antifungal drugs is riboflavin synthesis (6, 7). Riboflavin is considered a vitamin, as it is essential for human and animal growth, yet it has to be supplemented in the food and feed as no *de novo* synthesis occurs in these organisms. Riboflavin, or vitamin B_2_, serves as a precursor for flavin mononucleotide (FMN) and flavin adenine dinucleotide (FAD). Both coenzymes are necessary to form flavoproteins, which are involved in redox metabolism (8). The connection between FMN/FAD and iron-sulfur clusters, occurring in the electron transport chain, where reduction of Fe-S is accompanied by oxidation of FMN or FAD, is noteworthy (9). Commercial production of riboflavin is performed by microbial species, such as Bacillus subtilis and Candida famata (10, 11). Vitamin metabolism, and riboflavin production more specifically, has been investigated in terms of antimicrobial drug development. For organisms that cannot synthesize riboflavin themselves, such as *Lactobacilli* species, Enterococcus faecalis, and Listeria monocytogenes, riboflavin transport is indispensable (12). Other bacteria, such as Escherichia coli, *Salmonella*, and *Mycobacterium*, strictly depend on the endogenous production of riboflavin, as efficient transporter proteins are not available (13, 14). In these organisms, riboflavin synthase and lumazine synthase have been investigated thoroughly as attractive targets for antibacterial drug development. By slightly adapting the enzyme’s natural substrates, one can yield analogues, termed antimetabolites, which have an inhibitory effect on the enzyme’s activity (15). In this way, trifluoromethylated pyrazoles were found to inhibit the riboflavin synthase of Mycobacterium tuberculosis (16). Similarly, chemicals were found to inhibit the riboflavin synthase or lumazine synthase of Bacillus subtilis, E. coli, Brucella abortus, and others (17–19). Using a similar strategy, the laboratory of Markus Fisher has solved the crystal structure of two enzymes involved in riboflavin synthesis in C. albicans, more specifically Rib3 and Rib4 (20, 21). A number of inhibitors were modeled against the crystal structure of the enzymes, yet no *in vivo* evidence of inhibition was presented. Several enzymes encoded by *RIB* genes are involved in converting GTP and ribulose-5-phosphate into riboflavin and further into FMN and FAD. Many C. albicans genes are still uncharacterized and were assigned their name based on sequence homology with genes from the model organism Saccharomyces cerevisiae (22). The biosynthesis pathway of riboflavin, as established for S. cerevisiae, is depicted in Fig. S1 in the supplemental material (23, 24). C. albicans *RIB1* (*CaRIB1*), *CaRIB2*, *CaRIB4*, and *CaRIB7* are involved in conversion of GTP to 6,7-dimethyl-8-ribityllumazine. *CaRIB3* is involved in conversion of ribulose-5-phosphate to 3,4-dihydroxy-2-butanone 4-phosphate. *CaRIB5* is the last enzyme involved in the production of riboflavin. *CaFMN1* converts riboflavin into FMN.

10.1128/mSphere.00714-20.1 FIG S1 Schematic representation of the riboflavin biosynthesis pathway in S. cerevisiae. Based on homology with S. cerevisiae, C. albicans enzymes are depicted alongside the reactions, the enzymes for which the open reading frames are uncharacterized are shown in orange, and the others are in blue. More details are given in the text. This figure uses data from references 23 and 24. Download FIG S1, EPS file, 2.7 MB. Copyright © 2020 Demuyser et al. 2020 Demuyser et al. This content is distributed under the terms of the Creative Commons Attribution 4.0 International license.

In this report, we identify *Ca*Rib1 as a GTP cyclohydrolase by heterologous expression in S. cerevisiae and show that it catalyzes an essential and rate-limiting step in riboflavin synthesis. Deleting the gene completely impairs cytotoxicity against mammalian cells as well as infectibility in a systemic mouse model. We confirm that a low cellular iron content as well as high activity of protein kinase A (PKA) positively influence production of riboflavin (25) and show that expression of *CaRIB1* is elevated under both conditions. *Ca*Sef1, a transcription factor, regulates riboflavin synthesis in an iron- and PKA-dependent manner and is essential in a systemic mouse model of infection.

## RESULTS

### Riboflavin production can be measured through fluorescence emission of spent medium.

Riboflavin secreted to the culture medium was measured spectrophotometrically by analyzing fluorescence emission at 530 nm of the cell-free supernatant, with excitation at 450 nm. Figure S2 in the supplemental material shows the excitation and emission spectra of SC5314 culture supernatant (Fig. S2b) compared to pure riboflavin dissolved in the same medium (Fig. S2a). The standard curve depicted in Fig. S2c was used to quantify the riboflavin content as measured spectrophotometrically. All experiments in the manuscript were performed in LoFlo_glu_ medium mimicking complete synthetic medium with glucose but lacking riboflavin, unless stated otherwise.

10.1128/mSphere.00714-20.2 FIG S2 Fluorescence characteristics of riboflavin and culture supernatant. (a) Excitation and emission spectra of riboflavin, dissolved in LoFlo_glu_ medium, were determined. (b) Likewise, excitation and emission spectra of sterilized 24-h-old SC5314 culture supernatants were evaluated. (c) A standard curve of the fluorescence emission of riboflavin was set up. RFU, relative fluorescence units. Download FIG S2, EPS file, 1.5 MB. Copyright © 2020 Demuyser et al. 2020 Demuyser et al. This content is distributed under the terms of the Creative Commons Attribution 4.0 International license.

### *Ca*Rib1 is a central player in riboflavin synthesis.

**FIG 1:**
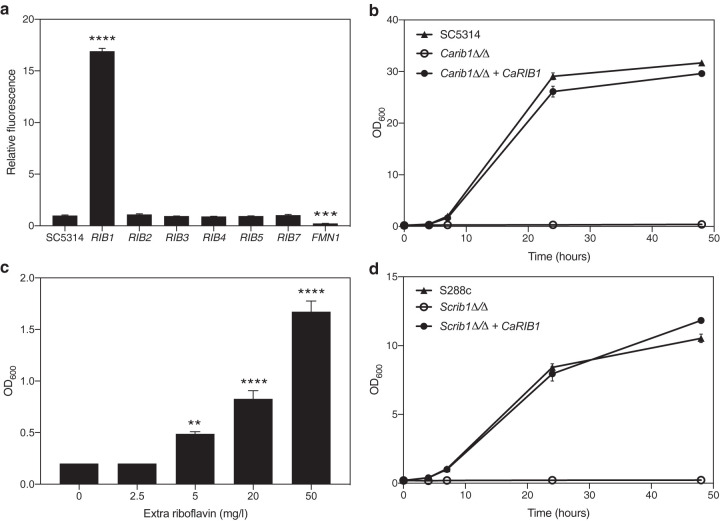
*Ca*Rib1 is a central regulator of riboflavin biosynthesis in C. albicans. (a) Strains overexpressing the genes encoding riboflavin synthesis enzymes were grown for 24 h, and fluorescence at 530 nm of the sterile supernatant was measured upon excitation at 450 nm. The relative ratio of fluorescence to OD_600_ is shown as average and SEM. Upon overexpression of *CaRIB1*, production of riboflavin increases. (b) Growth of the *Carib1*Δ/Δ strain as well as the reintegrant and the wild-type SC5314 strain was monitored over time. Deletion of *CaRIB1* inhibits growth in the absence of external riboflavin. Reintegration of the gene reverts this phenotype. (c) Growth of the *Carib1*Δ/Δ strain was assessed after 24 h in the absence and presence of external riboflavin. Data are shown as averages and SEM. Adding riboflavin partially restores the growth of this deletion strain. (d) Growth of the S. cerevisiae *rib1*Δ/Δ strain as well as the *CaRIB1* reintegrant and the wild-type S288c strain was monitored over time. *CaRIB1* is able to fully complement the S. cerevisiae orthologue in terms of growth in the absence of external riboflavin. Statistical analyses shown in panels a and c were performed by one-way analysis of variance (ANOVA), with Bonferroni correction. **, *P* < 0.01; ***, *P* < 0.001; ****, *P* < 0.0001.

To determine the rate-limiting enzymes in riboflavin biosynthesis, we generated strains overexpressing all eight genes putatively involved in the pathway. The laboratory reference SC5314 was used as the background strain. To objectively compare the effect of the increased dosage of the different genes, equal copy numbers of plasmid integration were verified. We used a setup based on quantitative PCR (qPCR) analysis, similar to that published by our laboratory earlier (26). Riboflavin production was monitored by fluorescence emission of the cell-free supernatant of cultures grown for 24 h. As shown in Fig. 1a, only overexpression of *CaRIB1* leads to an increase in the produced and secreted levels of riboflavin, indicating that Rib1 catalyzes the rate-limiting step of the production process. *CaRIB1* is an *ScRIB1* ortholog that is uncharacterized in C. albicans. It encodes a putative GTP cyclohydrolase II enzyme that converts GTP into 2,5-diamino-6-ribosylamino-4(H)-pyrimidinedione 5′-phosphate as one of the first steps in the riboflavin synthesis pathway (27). Overexpression of *CaFMN1* decreases riboflavin production, since this gene encodes a putative riboflavin kinase, converting riboflavin to the nonfluorescent flavin mononucleotide.

Deletion of both *CaRIB1* alleles in the SC5314 background strain completely inhibits growth in the absence of externally added riboflavin, indicating that it is an essential gene under conditions of low available riboflavin. Reintegration of the gene placed behind the constitutive *CaACT1* promoter reverts this growth deficit, as shown in Fig. 1b. Upon addition of increasing concentrations of riboflavin, growth of the *Carib1*Δ/Δ deletion strain is restored partially (Fig. 1c), indicating that a mechanism for riboflavin transport through the cell membrane must exist. Finally, we show that heterologous expression of *CaRIB1* can functionally complement the S. cerevisiae deletion strain, *Scrib1*Δ/Δ, as can be seen in Fig. 1d. This reinforces the role of *Ca*Rib1 as a GTP cyclohydrolase II involved in riboflavin synthesis.

### *Ca*Rib1 is essential for virulence in relevant model systems.

**FIG 2:**
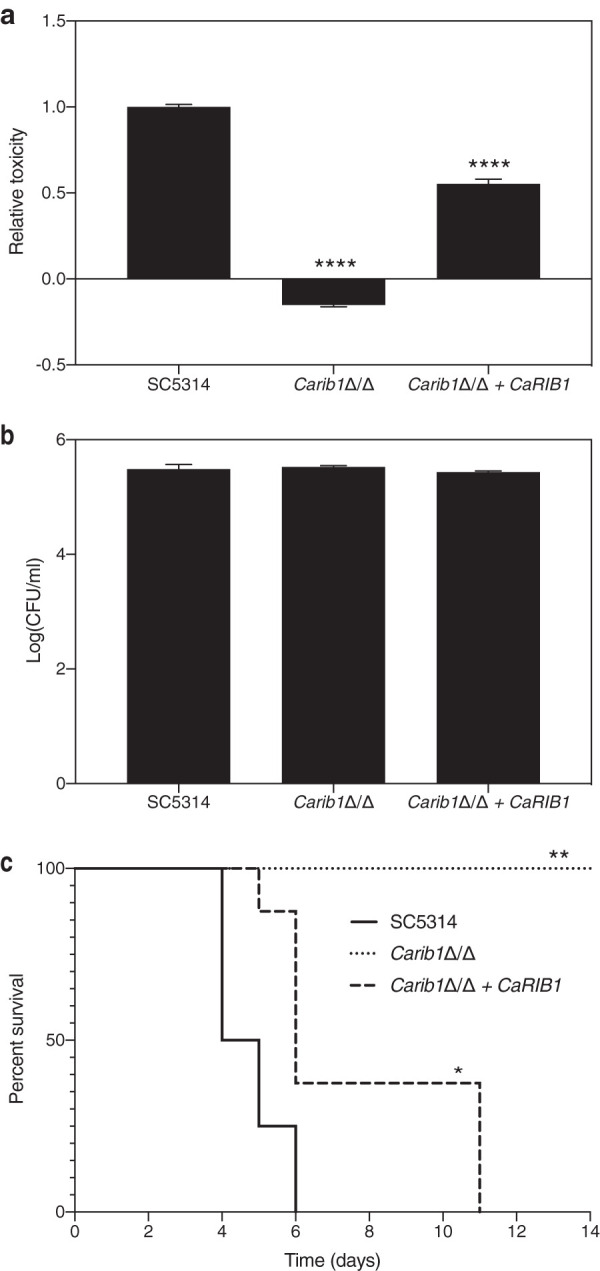
*Ca*Rib1 is essential for full virulence in relevant model systems. (a) The *Carib1*Δ/Δ deletion strain as well as the wild-type and the reintegrant strains were added to mammalian HeLa cells. After 24 h of incubation at 37°C and 5% CO_2_, cytotoxicity was determined based on LDH release in the culture supernatant. Deletion of *CaRIB1* causes the complete absence of cytotoxicity toward mammalian HeLa cells (determined by LDH activity and shown as average and SEM). (b) The *Carib1*Δ/Δ deletion, wild-type, and reintegrant strains were added to mammalian HeLa cells. After 90 min of incubation at 37°C and 5% CO_2_, adhesion was determined based on number of CFU. Average values and SEM are shown. There is no significant difference between the deletion strain and the wild type. (c) Mice were infected with the same strains through tail vein injection. The C. albicans strain without *Ca*Rib1 is completely avirulent in a mouse model of systemic infection. Statistical analyses for panels a and b were performed by one-way ANOVA, with Bonferroni correction. ****, *P* < 0.0001. Statistical analysis for panel c was performed by a log rank Mantel-Cox test. *, *P* < 0.05; **, *P* < 0.01.

*CaRIB1* is essential under conditions where low levels of external riboflavin are present. The concentration of this vitamin in human serum ranges between 0.04 and 0.24 mg/liter, which is significantly lower than the concentration needed for *in vitro* growth restoration of the deletion strain (28). To determine whether Rib1 is also essential in a context resembling the *in vivo* situation, we evaluated the effect of its deletion in the laboratory strain on toxicity toward mammalian HeLa cells. Toxicity is measured by spectrophotometric analysis of lactate dehydrogenase (LDH) release in the cell supernatant. Figure 2a shows that the *Carib1*Δ/Δ deletion strain imposes no toxicity toward the HeLa cells, while reintegration of the gene partially restores this phenotype. We confirm that the reduced toxicity is not caused by a reduction of microbial cell adhesion to the mammalian cells (Fig. 2b).

The inability of the *Carib1*Δ/Δ deletion strain to grow in the absence of adequate amounts of riboflavin is confirmed in a mouse model of systemic infection. Mice were infected with 8.5 × 10^5^ cells per mouse by intravenous injection and were inspected daily for morbidity and mortality. Upon signs of severe morbidity, such as over 15% weight loss, the mice were sacrificed. As can be seen in Fig. 2c, the strain lacking both *CaRIB1* alleles is completely avirulent in this mouse model, while the wild-type and reintegrant strains start causing mortality after 4 or 5 days, respectively. These results indicate that *Ca*Rib1 is a promising novel antifungal drug target.

### The iron content of the growth medium modulates riboflavin production.

**FIG 3:**
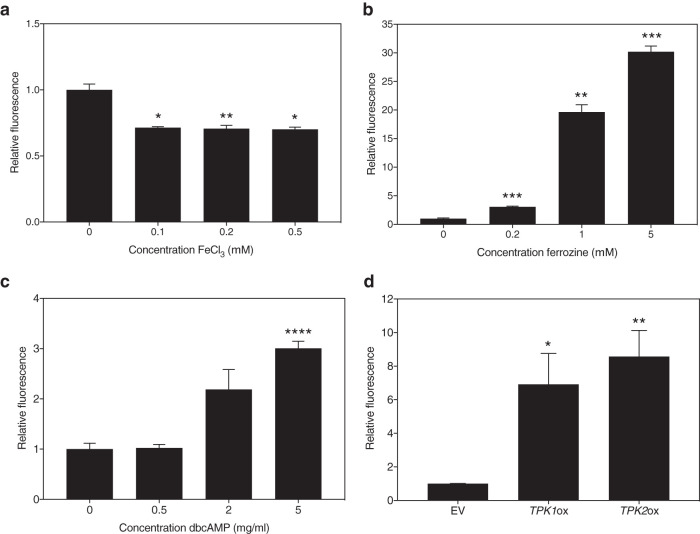
Riboflavin production alters upon iron addition or sequestration and PKA activation. SC5314 cultures were grown for 24 h in the presence of FeCl_3_ (a), an iron chelator, ferrozine (b), or dbcAMP (c). (d) *CaTPK1* and *CaTPK2* overexpression strains were grown for 24 h, as well as the empty vector (EV) control strain. The fluorescence was measured at 530 nm when excited upon 450 nm. The average and the SEM of relative fluorescence to OD_600_ ratios are shown. Statistical analysis was performed using a one-way analysis of variance (ANOVA) statistical test with Bonferroni correction (comparison to culture without added compound). *, *P* < 0.05; **, *P* < 0.01; ***, *P* < 0.001; ****, *P* < 0.0001.

A connection between flavin metabolism and iron was established earlier, as FAD and FMN are involved in the redox state of Fe-S clusters (9). To determine the effect of iron on the production or secretion of riboflavin to the medium, we cultured the SC5314 laboratory strain in the presence of different concentrations of FeCl_3_ or Fe_2_(SO_4_)_3_ and iron chelator bathophenanthrolinedisulfonate (BPS) or ferrozine. As can be seen in Fig. 3a and Fig. S3a, the addition of iron diminishes the riboflavin content in the culture supernatant. Addition of increasing concentrations of ferrozine or BPS has the opposite effect, as shown visually (Fig. S4) and quantitatively (Fig. 3b and Fig. S3b). Thus, we can conclude that iron levels negatively correlate with production of riboflavin in C. albicans.

10.1128/mSphere.00714-20.3 FIG S3 Riboflavin production alters upon iron addition or sequestration. SC5314 cultures were grown for 24 h in the presence of Fe_2_(SO_4_)_3_ (a) or an iron chelator, BPS (b). The fluorescence was measured at 530 nm when excited upon 450 nm. The average and the SEM of relative fluorescence to OD_600_ ratios are shown. Statistical analysis was performed using a one-way analysis of variance (ANOVA) statistical test with Bonferroni correction (comparison to culture without added compound). *, *P* < 0.05; **, *P* < 0.01. Download FIG S3, EPS file, 1.3 MB. Copyright © 2020 Demuyser et al. 2020 Demuyser et al. This content is distributed under the terms of the Creative Commons Attribution 4.0 International license.

10.1128/mSphere.00714-20.4 FIG S4 Empirical observation of riboflavin production in the presence of iron chelators. SC5314 cultures were grown for 24 h in the presence of 0, 0.2, 1, and 5 mM ferrozine (a) or 0, 0.02, 0.05, and 0.1 mg/ml BPS (c). Blank medium with chelator was incubated to compare ferrozine (b) and BPS (d). Images were taken of the cultures (upper), and cultures were spun down and imaged using blue light at 460 nm (lower). Download FIG S4, TIF file, 2.6 MB. Copyright © 2020 Demuyser et al. 2020 Demuyser et al. This content is distributed under the terms of the Creative Commons Attribution 4.0 International license.

### Riboflavin production and autofluorescence increase upon PKA stimulation.

Activity of the cyclic AMP (cAMP)-PKA pathway has been linked to riboflavin production in certain fungi (29, 30). We confirm that increasing signaling through this pathway augments the amount of riboflavin found in the culture supernatant of the C. albicans wild-type strain. Figure 3c shows the effect of adding increasing concentrations of dibutyryl cAMP (dbcAMP) to the growth medium of the SC5314 wild-type strain. This compound is an analogue of cAMP and activates PKA. We also generated mutant strains overexpressing the genes encoding either of the catalytic PKA subunits of C. albicans, *Ca*Tpk1 and *Ca*Tpk2. We verified equal amounts of copy number insertions of the overexpression plasmids. Both *CaTPK1* and *CaTPK2* overexpression strains showed increased amounts of riboflavin in the culture supernatant after 24 h of growth (Fig. 3d). Both results indicate that PKA activity positively affects riboflavin production.

Riboflavin is a naturally fluorescent product, excitable at 450 nm and emitting light at a wavelength of 530 nm. As it is of importance in fluorescence experimentation, we verified whether increased production of riboflavin, through overactivation of the cAMP-PKA signaling pathway, would have an effect on the autofluorescence level of the cells. As shown in Fig. S5a, addition of increasing amounts of dbcAMP significantly increases the cellular fluorescence at a wavelength of 531/40 nm, as measured by flow cytometry (excitation at 488 nm). We also showed increasing autofluorescence levels in the *CaTPK1* and *CaTPK2* overexpression strains (Fig. S5b).

10.1128/mSphere.00714-20.5 FIG S5 Autofluorescence levels alter upon activation of the cAMP-PKA pathway. (a) SC5314 cultures were grown for 24 h in the presence of dbcAMP. (b) *CaTPK1* and *CaTPK2* overexpression strains were grown for 24 h, as was the empty vector (EV) control strain. The fluorescence was measured at 531/40-nm emission when excited with a 488-nm light. The average and the SEM of relative fluorescence to OD_600_ ratios are shown. Statistical analysis was performed using a one-way ANOVA statistical test with Bonferroni correction (comparison to culture without added compound or EV strain). RFU, relative fluorescence units. *, *P* < 0.05; **, *P* < 0.01; ***, *P* < 0.001. Download FIG S5, EPS file, 1.3 MB. Copyright © 2020 Demuyser et al. 2020 Demuyser et al. This content is distributed under the terms of the Creative Commons Attribution 4.0 International license.

### Iron deprivation and PKA overactivation alter transcription of *CaRIB* synthesis genes.

We determined whether activation of cAMP-PKA signaling and iron deprivation could cause increased production of riboflavin via transcriptional regulation. To do so, we assessed gene expression of several riboflavin synthesis-associated genes in the presence of an iron chelator in the SC5314 wild-type strain as well as in *CaTPK1* and *CaTPK2* overexpression strains.

**FIG 4:**
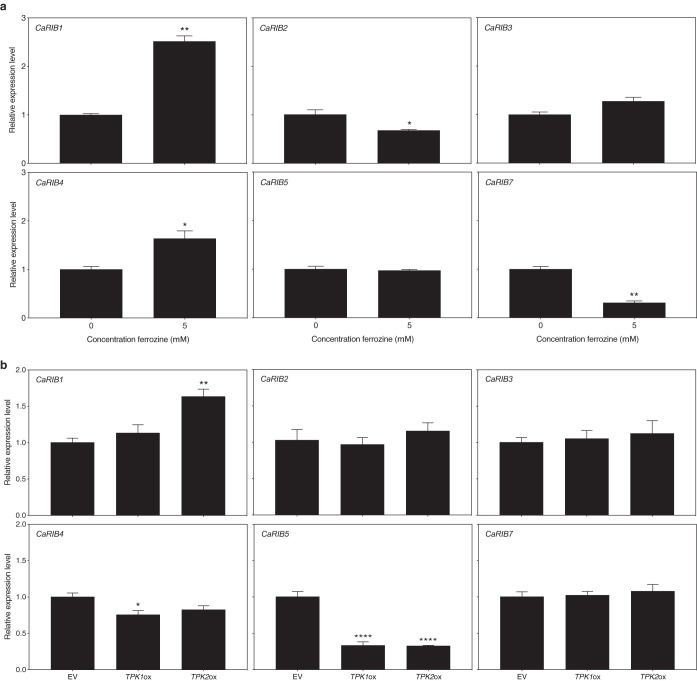
Expression of genes involved in riboflavin biosynthesis alters upon iron deprivation and PKA activation. (a) SC5314 cultures were grown for 8 h in the presence of ferrozine. (b) The empty vector (EV) control strain and *CaTPK1* and *CaTPK2* overexpression cultures were grown for 8 h. Gene expression was analyzed using qRT-PCR. Results are displayed as the average and SEM relative to the wild-type control. Statistical analysis was conducted on log_2_(Y)-transformed data using one-way ANOVA with Bonferroni correction. *, *P* < 0.05; **, *P* < 0.01; ****, *P* < 0.0001.

Figure 4a shows the effect of an iron chelator, ferrozine, on the expression of the main riboflavin biosynthesis enzyme-encoding genes. We show that expression of *CaRIB1* and *CaRIB4* is upregulated upon iron deprivation. This was confirmed by using the alternative iron chelator BPS (Fig. S6). As mentioned earlier, the open reading frame (ORF) of *CaRIB1* was uncharacterized, yet the orthologue in S. cerevisiae is known to encode the first enzyme in riboflavin biosynthesis, a GTP cyclohydrolase II (27). *CaRIB4* encodes the lumazine synthase (31). *CaRIB7* is uncharacterized as well, and the S. cerevisiae orthologue encodes the 5-amino-6-(5-phosphoribosylamino)uracil reductase (27). In the presence of ferrozine and BPS, the expression of this gene is downregulated. In the presence of ferrozine, expression of *CaRIB2* also is downregulated (Fig. 4a); however, this is not the case in the presence of BPS (Fig. S6). The S. cerevisiae orthologue of *CaRIB2* encodes a 2,5-diamino-6-ribitylamino-4(3H)-pyrimidinone 5′-phosphate deaminase enzyme (27). Figure 4b shows the effect of PKA activation via *CaTPK* overexpression on the expression of the main riboflavin biosynthesis enzyme-encoding genes. Only expression of *CaRIB1* was significantly upregulated in the *CaTPK2* overexpression strain. Remarkably, expression of *CaRIB5* was significantly downregulated in both overexpression strains. *CaRIB1* expression is increased upon both iron limitation and PKA activation. This indicates that *Ca*Rib1 is involved in riboflavin synthesis as a central point of regulation.

10.1128/mSphere.00714-20.6 FIG S6 Expression of genes involved in riboflavin biosynthesis alters upon iron deprivation. SC5314 cultures were grown for 8 h in the presence of BPS. Gene expression was analyzed using qRT-PCR. Results are displayed as the average relative to the wild-type control and together with the SEM. Statistical analysis was conducted on log_2_(Y)-transformed data using one-way ANOVA with Bonferroni correction. *, *P* < 0.05; **, *P* < 0.01. Download FIG S6, EPS file, 1.8 MB. Copyright © 2020 Demuyser et al. 2020 Demuyser et al. This content is distributed under the terms of the Creative Commons Attribution 4.0 International license.

### *Ca*Sef1 regulates riboflavin synthesis in an iron- and PKA-dependent manner.

**FIG 5:**
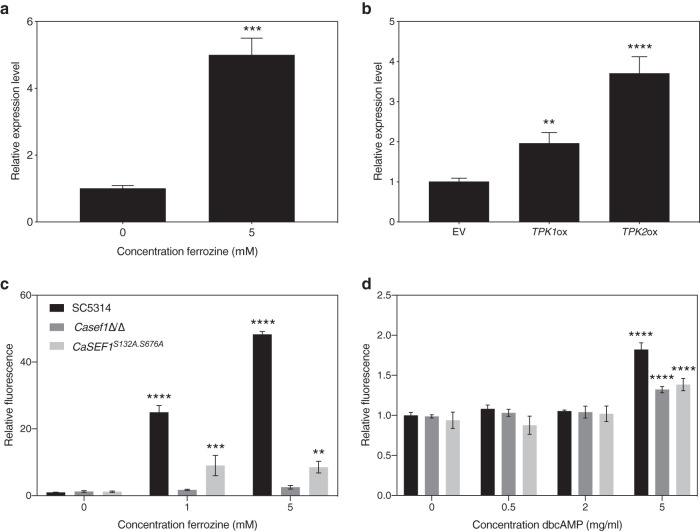
*Ca*Sef1 and its phosphorylation are necessary for riboflavin production in the absence of iron and presence of PKA activation. (a) SC5314 cultures were grown for 8 h in the presence of ferrozine. (b) The empty vector (EV) control strain and *CaTPK1* and *CaTPK2* overexpression cultures were grown for 8 h. Gene expression was analyzed using qRT-PCR. Results are displayed as the average and SEM relative to the wild-type control. Statistical analysis was conducted on log_2_(Y)-transformed data using one-way ANOVA with Bonferroni correction. SC5314, *Casef1*Δ/Δ, the *Ca*Sef1 phosphorylation mutant, and the reintegrant strain were grown for 24 h in the presence of ferrozine (c) or dbcAMP (d). The fluorescence was measured at 530 nm when excited upon 450 nm. The average and SEM of relative fluorescence to OD_600_ ratios are shown. Statistical analysis was performed using a two-way ANOVA statistical test with Bonferroni correction (comparison to culture without added compound). **, *P* < 0.01; ***, *P* < 0.001; ****, *P* < 0.0001.

*CaSEF1* encodes a Zn2-Cys6 transcription factor that is reported to be involved in the regulation of iron uptake (32). In other organisms, Sef1 is also involved in regulation of riboflavin synthesis (8). This transcription factor was shown to play an essential role in regulation of *RIB* genes, although the exact mechanisms are not known yet (32, 33). We show that the expression of the gene encoding the transcriptional regulator *Ca*Sef1 is strongly induced under iron deprivation conditions, as presented in Fig. 5a. Expression of *CaSEF1* was also significantly upregulated in both *CaTPK1* and *CaTPK2* overexpression strains, as can be seen in Fig. 5b.

To confirm the involvement of Sef1 in riboflavin synthesis in C. albicans and to verify whether this is modulated by iron levels and PKA activity, we compared riboflavin production in a *Casef1*Δ/Δ deletion strain to that of the SC5314 wild type in the presence of dbcAMP or ferrozine. As can be seen in Fig. 5c and d, respectively, deletion of *CaSEF1* has no effect on riboflavin production in the absence of iron chelation or PKA stimulation. However, in the presence of low iron levels or upon PKA activation, the deletion strain is unable to synthesize as much riboflavin as the wild-type strain. This indicates that Sef1 is an essential link between the upstream input signals, iron limitation, and PKA activation and the downstream response, riboflavin synthesis.

We discovered, using the ScanSite tool (34), that *Ca*Sef1 contains two putative PKA phosphorylation sites, S132 and S676, and generated a mutant strain in which both serines are mutated to alanines, thereby preventing phosphorylation. Figure 5c and d show that this strain does produce significantly less riboflavin than the wild-type strain, indicating that the hypothesized phosphorylation of *Ca*Sef1 by PKA is essential under both PKA-activating as well as iron-limiting conditions.

### *Ca*Sef1 but not its phosphorylation by PKA is essential for virulence in a mouse model of systemic infection.

**FIG 6:**
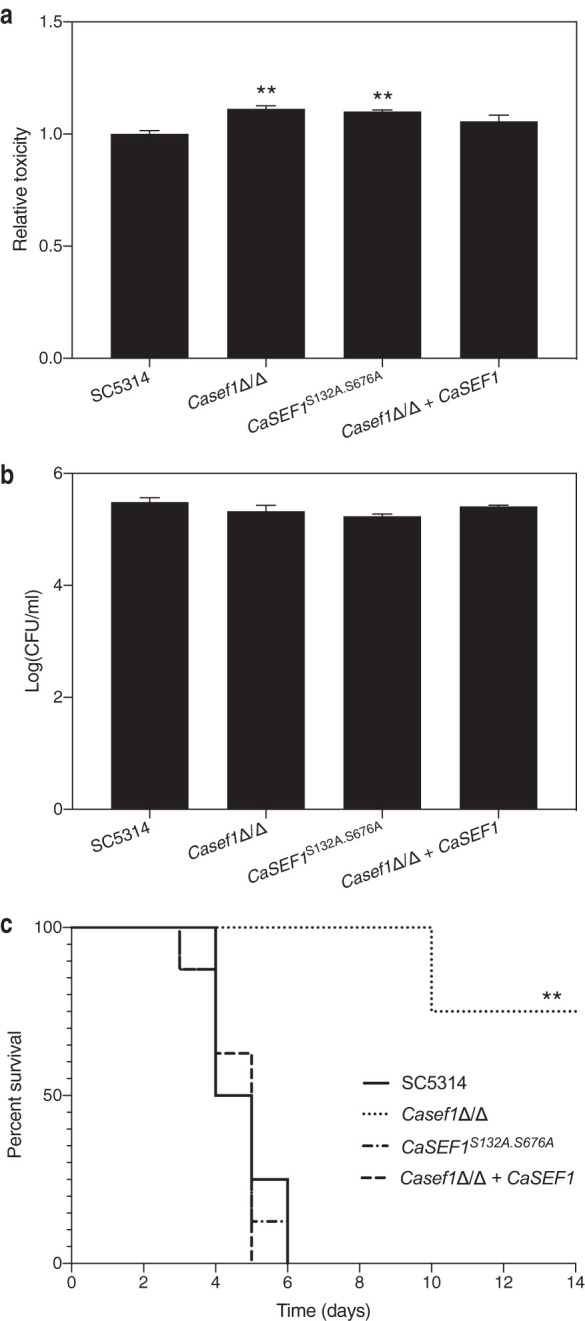
*Ca*Sef1 is essential for full virulence in a systemic mouse model of infection. (a) The *Casef1*Δ/Δ deletion strain as well as the wild-type, the phosphorylation mutant, and the reintegrant strains were added to mammalian HeLa cells. After 24 h of incubation at 37°C and 5% CO_2_, cytotoxicity was determined based on LDH release in the culture supernatant. Data are shown as average and SEM. Neither deletion nor mutation of *CaSEF1* causes a decrease of cytotoxicity toward mammalian HeLa cells (determined by LDH activity). (b) The *Casef1*Δ/Δ deletion, phosphorylation mutant, wild-type, and reintegrant strains were added to mammalian HeLa cells. After 90 min of incubation at 37°C and 5% CO_2_, adhesion was determined based on number of CFU. Average values and SEM are shown. There is no significant difference between the mutant strains and the wild type. (c) Mice were infected with the same strains through tail vein injection. The C. albicans strain without *Ca*Sef1 is less virulent in a mouse model of systemic infection. Statistical analyses of panels a and b were performed by one-way ANOVA, with Bonferroni correction. **, *P* < 0.01. Statistical analysis of panel c was performed by a log-rank Mantel-Cox test. **, *P* < 0.01.

It was shown earlier that *Ca*Sef1 is essential under iron-limiting conditions (32). To determine whether this transcription factor is also essential for full virulence in an *in vivo* context, we assessed toxicity against mammalian cells, imposed by the mutant strain compared to the wild-type SC5314 strain, as described earlier. Figure 6a reveals that neither the deletion strain nor the phosphorylation mutant show a decrease in toxicity imposed on HeLa cells. This indicates that neither *Ca*Sef1 nor its putative phosphorylation by PKA is essential for virulence in this model. Figure 6b confirms that no difference in adhesion to the mammalian cells can be detected.

Since researchers reported earlier that the *Casef1*Δ/Δ deletion strain is required for full virulence in a mouse model of systemic infection, we tested this strain as well as the phosphorylation mutant in a similar model system (32). Figure 6c indeed shows that deleting both alleles of the *CaSEF1* gene impairs full virulence in mice and that reintegration of the gene behind the constitutive *CaACT1* promoter restores this phenotype. However, preventing putative phosphorylation by PKA at sites S132 and S676 does not alter virulence compared to that of the wild-type SC5314 strain. This implies that phosphorylation of *Ca*Sef1 at these specific sites does not play a major role in the *in vivo* context that we tested.

## DISCUSSION

In the manuscript, we show that riboflavin production is a promising target for development of novel drugs against C. albicans. *Ca*Rib1 plays a central role in this process. By heterologous expression in S. cerevisiae, we confirmed the function of this enzyme in the riboflavin synthesis process, being a GTP cyclohydrolase. Deletion of this gene renders the strain nonviable in the absence of externally added riboflavin. Upon addition of riboflavin to the culture medium, the strain can be rescued, indicating that transport is possible. Thus, the potential of using *Ca*Rib1 as a drug target depends on the concentration of riboflavin that is present in the host niche where C. albicans is expected to be infectious. Based on the literature, it can be stated that the concentration of riboflavin in human blood are significantly lower than the minimal concentration needed to sustain growth *in vitro*. This is confirmed by our *in vivo* data, where we show that the *Carib1*Δ/Δ deletion strain is not able to cause any morbidity or mortality in a systemic mouse model of infection. Whether the concentration of riboflavin is adequate in other host niches and whether targeting *Ca*Rib1 would also be promising in other types of infections remain to be investigated. Apart from the essential character of *Ca*Rib1, this enzyme is interesting as an antifungal drug target in another respect as well. Upon overexpression of all *RIB* genes separately and measurement of the effect on riboflavin production, only overexpression of *CaRIB1* leads to an increase in production, indicating that *Ca*Rib1 catalyzes a rate-limiting step in the process. Thus, targeting *Ca*Rib1 is thought to be more effective than targeting any of the other enzymes.

**FIG 7:**
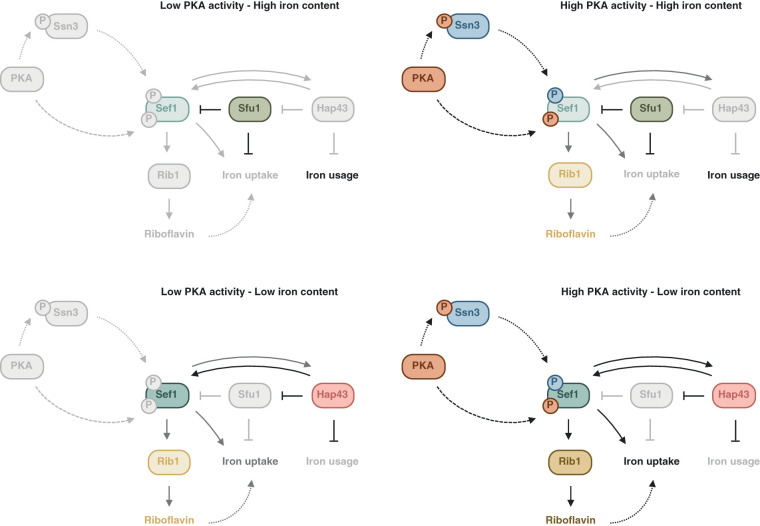
Schematic representation of hypothetical iron- and PKA-dependent regulation of riboflavin synthesis. For four situations, high/low iron and high/low PKA activity, the hypothetical regulation of riboflavin synthesis is illustrated based on data from the manuscript as well as Chen et al. (32). Details can be found in the text. The higher the intensity/darkness of the depicted symbol or arrow, the higher the activity of the enzyme or reaction. Full arrows depict known interactions, striped arrows depict hypothetical, partially verified interactions, and dotted arrows depict purely hypothetical interactions. P, phosphate; PKA, protein kinase A.

Upstream regulation of riboflavin production is not fully investigated in C. albicans. However, given the established importance of *Ca*Rib1 in the production process, it would be reasonable that upstream regulation targets this essential and rate-limiting factor in the process. We show that this is indeed the case. It was established earlier that iron deficiency stimulates production of riboflavin (25). We confirm this observation and show that under these conditions, expression of *CaRIB1* is upregulated. It has been hypothesized by others that riboflavin acts as a type of siderophore important in the uptake of iron (8). This would explain why it is secreted in the culture medium, as it would have to scavenge iron externally. Whether this is indeed the case, however, will have to be investigated further. We also demonstrate that activating PKA increases riboflavin synthesis and, thereby, cellular autofluorescence. These findings have practical implications for cell biologists who seek to visualize green fluorescent protein-labeled proteins, as the wavelengths we used for excitation and emission detection of autofluorescence are the approximate wavelengths of this often-used fluorophore. Caution should be taken when performing quantitative fluorescence microscopy with C. albicans under PKA-altering conditions. Since *CaRIB1* expression is upregulated upon activation of PKA, it is likely that regulation of riboflavin production happens mainly through *Ca*Rib1. A possible factor linking upstream inducing factors and *CaRIB1* gene expression is the transcription factor *Ca*Sef1. This transcriptional regulator has been implicated in regulation of riboflavin synthesis in other organisms (8). We show that deletion of both alleles of the *CaSEF1* gene impairs overproduction of riboflavin in the presence of an iron chelator or PKA activator. This indicates that the transcription factor is essential in signaling upstream cues, such as iron limitation or PKA activation, down to the riboflavin biosynthesis enzymes. We also noticed that under these specific conditions, expression of *CaSEF1* itself is upregulated. It was reported earlier that *Ca*Sef1 can indeed regulate its own expression in a feedback loop through *Ca*Hap43 and *Ca*Sfu1, two other transcription factors involved in regulation of iron uptake (32). It has been shown as well that *Ca*Sef1 is activated under iron-limiting conditions. The mechanism by which PKA activity could be linked to *Ca*Sef1 remained largely elusive. We can discern two hypothetical models for PKA to affect riboflavin synthesis. A first way in which PKA can influence riboflavin production is indirect by affecting iron uptake in the cell. It was shown for S. cerevisiae that *Sc*Tpk2 negatively regulates iron uptake (35). Therefore, the overexpression of this gene could mimic iron deficiency. Second, it is possible that PKA influences the synthesis of this metabolite. Using the online protein motif search tool ScanSite to search for putative phosphorylation sequences in *Ca*Sef1, we found two potential PKA-specific phosphorylation sequences (34). Direct phosphorylation of *Ca*Sef1 would present a possible regulatory mechanism of riboflavin synthesis by PKA. We generated a mutant strain in which *Ca*Sef1 has two phosphorylatable serines replaced by alanines. *In vitro* data show that PKA phosphorylation could indeed be essential for its function, as the *CaSEF1^S132A.S676A^* mutant strain resembles the deletion mutant to a large extent. However, our *in vivo* data do not support this conclusion. The *CaSEF1^S132A.S676A^* mutant strain resembles the wild type and imposes high levels of virulence on the mice. It is possible, though, that other *in vivo* model systems show alternative results or that other phosphorylation sites exist. Another potential link between PKA and *Ca*Sef1 includes the protein kinase *Ca*Ssn3. PKA-regulated activation of *Ca*Ssn3 as well as *Ca*Ssn3-regulated activation of *Ca*Sef1 have been reported for C. albicans (36, 37). Figure 7 schematically depicts the hypothetical model of how riboflavin production can be regulated by iron availability as well as PKA activity. Further experimentation is necessary to unravel the exact regulation of riboflavin synthesis.

The role of riboflavin production in microbial pathogenesis has been recognized relatively recently. It was shown for several pathogens, including C. albicans, that the production of this vitamin in nutrient-poor host niches is essential for survival and, thus, is important for virulence (6, 38–40). As riboflavin production is an essential process in C. albicans and no orthologues of the enzymes involved are present in human cells, riboflavin production is an attractive target for new antifungal drugs (6, 7).

## MATERIALS AND METHODS

### Strains and plasmids.

The C. albicans strains used in this study are listed in Table S1 in the supplemental material. Plasmids are summarized in Table S2 and primers in Table S3. For constructing overexpression and reintegrant C. albicans strains, *CaTPK1*, *CaTPK2*, *CaSEF1*, and *CaRIB* genes and *CaFMN1* were cloned in the integrative plasmid CIp10-*NAT1* (26, 41). *CaTPK1* was integrated in the NheI-ClaI-cut Clp10-*NAT1* vector, while *CaTPK2*, *CaSEF1*, *CaFMN1*, and all *CaRIB* genes were integrated in the NheI-AatII-cut Clp10-*NAT1* vector. For integration, plasmids and the empty plasmid CIp10-*NAT1* were linearized by StuI and transformed using a lithium acetate method (42). All overexpression strains, *CaTPK1*ox, *CaTPK2*ox, *CaRIB*ox, and *CaFMN1*ox, and the empty vector control strain (EV) were generated in the SC5314 background. Integration was verified by diagnostic PCR. For each transformation, we obtained 4 independent transformants with equal copy number insertions. C. albicans *rib1*Δ/Δ and *sef1*Δ/Δ deletion strains as well as the *CaSEF1^S132A.S676A^* phosphorylation mutant were constructed in the background strain SC5314 using the CRISPR system described by Nguyen et al. (43). With their HIS-FLP split marker system, gRNA expression cassettes were amplified from plasmids pADH110 and pADH147 and cotransformed with plasmid pADH99 (*CAS9* harboring) after digestion by MssI. *Carib1*Δ/Δ and *Casef1*Δ/Δ mutants were constructed with the guide RNAs 5′-TACATTGGTAGATTGACACC and 5′-TTACAATTAGGTCTACATCG, respectively. Duplexed oligonucleotide donor DNA (89 bp), comprising upstream and downstream sequences of the targeted gene, were provided as repair templates. For constructing the *CaSEF1^S132A.S676A^* phosphorylation mutant, serine (TCA) to alanine (GCC) mutations were introduced simultaneously using the guide RNAs 5′-TGGATTTGTGACCCTTTGCG and 5′-AAGACGATCAGTACTTGATA, targeting S132 and S676, respectively. Two 89-bp duplexed oligonucleotides containing the desired GCC mutation and 43-bp upstream and downstream homologous flanking sites were used simultaneously as donor DNA, generating strain *CaSEF1^S132A.S676A^.* After verifying the modification with diagnostic PCR, the *NAT1* marker and CRISPR components were removed by induction of the FLP recombinase in YP maltose (2%). All deletions and mutations were confirmed by Sanger sequencing (Eurofins Genomics). By integrating plasmid CIp10-*CaRIB1-NAT1* or CIp10-*CaSEF1-NAT1* in their respective deletion strain, reintegrant strains *Carib1*Δ/Δ + *CaRIB1* and *Casef1*Δ/Δ + *CaSEF1* were constructed. Integration was verified by diagnostic PCR. For each transformation, we obtained at least 3 independent transformants with equal copy number insertions. For heterologous expression of *CaRIB1* in the diploid S. cerevisiae strain S288c, a stepwise CRISPR/Cas9-based replacement methodology was applied. The *CAS9*- and gRNA-expressing plasmids were created by DiCarlo et al. and modified by Cen and coworkers (44, 45). First, both *ScRIB1* alleles were replaced by deletion cassettes constitutively expressing dominant hygromycin and nourseothricin resistance markers and flanked by G1 gRNA recognition sites. The selection markers were amplified from plasmids pTOPO-G1-*HPH*-G1 and pTOPO-G1-*NatMX*-G1, with primers containing tails homologous to the *ScRIB1* flanking regions. Correct integration of the cassettes was confirmed by PCR. Next, *CAS9*-expressing plasmid pTEF-*CAS9*-*BLE* was transformed. A G1-specific protospacer was assembled into pgRNA-uni-*KanMX* with the NEBuilder HiFi DNA assembly cloning kit (New England Biolabs) by following the manufacturer’s instructions. The design of this gRNA was based on the finding of Farboud and Meyer that Cas9-mediated DNA cleavage was enhanced at this G1 site (5′-GGCTGATTTTCGCAGTTCGGGGG) (46). This gRNA was cotransformed together with the appropriate donor DNA. To construct *Scrib1*Δ/Δ, donor DNA contained 89-bp duplexed oligonucleotide donor DNA, comprising upstream and downstream sequences. For heterologous expression of *CaRIB1* in S288c, donor DNA was PCR amplified from SC5314 genomic DNA, with primers containing tails homologous to sequences upstream and downstream of *ScRIB1*. gRNA- and *CAS9*-expressing plasmids were lost by propagation under nonselective conditions. The genotype of transformants was confirmed by Sanger sequencing (Eurofins Genomics). C. albicans and S. cerevisiae strains were propagated in YPD (1% yeast extract [Merck], 2% Bacto peptone [Oxoid], 2% dextrose). For solid media, 2% Difco granulated agar was added. Where required, 200 mg/liter riboflavin (Sigma) was added. For selecting C. albicans transformants, 200 mg/liter nourseothricin (Jena Bioscience) was added. For transforming S. cerevisiae, 100 mg/liter nourseothricin (Jena Bioscience), 300 mg/liter hygromycin B (InvivoGen), 10 mg/liter phleomycin (InvivoGen), and/or 200 mg/liter Geneticin G418 sulfate (ThermoFisher) were added.

10.1128/mSphere.00714-20.7 TABLE S1 Strains used in this study. Download Table S1, DOCX file, 0.02 MB. Copyright © 2020 Demuyser et al. 2020 Demuyser et al. This content is distributed under the terms of the Creative Commons Attribution 4.0 International license.

10.1128/mSphere.00714-20.8 TABLE S2 Plasmids used in this study. Download Table S2, DOCX file, 0.02 MB. Copyright © 2020 Demuyser et al. 2020 Demuyser et al. This content is distributed under the terms of the Creative Commons Attribution 4.0 International license.

10.1128/mSphere.00714-20.9 TABLE S3 Primers used in this study. Download Table S3, DOCX file, 0.02 MB. Copyright © 2020 Demuyser et al. 2020 Demuyser et al. This content is distributed under the terms of the Creative Commons Attribution 4.0 International license.

### Growth conditions: media and chemicals.

C. albicans strains were grown in LoFlo_glu_ medium. This medium contains 0.079% complete supplement mixture (CSM; MP Biomedicals), 0.19% yeast nitrogen base without amino acids and lacking folic acid and riboflavin (Formedium), 0.5% ammonium sulfate (Fluka), and 2% glucose. The pH was adapted to 5.5 in liquid medium. For some experiments, the growth medium of the wild-type strain SC5314 was supplemented with different concentrations of FeCl_3_ (Sigma), Fe_2_(SO_4_)_3_ (Sigma), BPS (Sigma), ferrozine (Sigma), or dibutyryl-cAMP (Sigma).

### Riboflavin measurement in cell supernatant.

C. albicans strains were grown overnight at 30°C in 3 ml LoFlo_glu_ medium. The overnight cultures were adapted to an optical density at 600 nm (OD_600_) of 0.1 in 3 ml LoFlo_glu_ medium and grown at 30°C. In selected experiments, FeCl_3_ (0.1, 0.2, and 0.5 mM), Fe_2_(SO_4_)_3_ (0.1, 0.2, and 0.5 mM), BPS (0.02, 0.05, and 0.1 mg/ml), ferrozine (0.2, 1, and 5 mM), or dbcAMP (0.5, 2, and 5 mg/ml) was added to the new medium. After 24 h of growth, 1 ml of the cell cultures was transferred to Eppendorf tubes and the OD_600_ was measured. After centrifugation, 0.8 ml of the cell supernatant was transferred to a new tube. The centrifugation step was repeated, and 0.6 ml cell supernatant was retrieved and used for further analysis in the Synergy H1 hybrid multimode microplate reader (BioTek). Wells of a black-walled 96-well microtiter plate (Greiner) were filled with 0.2 ml cell supernatant, and absorbance at 445 nm was measured. Fluorescence measurements were performed using emission at 530 nm upon excitation at 450 nm. For all quantitative experiments, the ratio of this fluorescence to the OD_600_ is shown. In some of the experiments, the presence of riboflavin in the culture supernatant was imaged using blue light of 460 nm.

### Autofluorescence measurement by FACS.

C. albicans strains were grown overnight at 30°C in 3 ml LoFlo_glu_ medium. The overnight cultures were adapted to an OD_600_ of 0.1 in 3 ml LoFlo_glu_ medium and grown at 30°C. For the wild-type SC5314 strain, dbcAMP (2 and 5 mg/ml) was added to the new medium. After 24 h the cells were washed 3 times and adjusted to an OD_600_ of 1 in LoFlo_glu_ medium. Flow cytometry was performed using the BD influx flow cytometer. The samples were analyzed using a blue laser (488 nm) in combination with an emission filter of 531/40 nm.

### Copy number determination by qPCR.

After transformation of SC5314 with the overexpression plasmids, genomic DNA was extracted from the transformants. Quantitative PCR (qPCR) was performed using GoTaq polymerase (Promega) and the StepOnePlus real-time PCR device (ThermoFisher). qPCRs were conducted on 2.5 ng genomic DNA per reaction, with primers for the *CaACT1* promoter region of the CIp10 plasmid and *Ca18S*, *CaTEF1*, and *CaACT1* as reference genes. Primers used for copy number determination are listed in Table S3. qBasePlus software (Biogazelle) was used for copy number analysis.

### RNA extraction and gene expression analysis by qRT-PCR.

C. albicans strains were grown overnight at 30°C in 5 ml LoFlo_glu_ medium. The overnight cultures were adapted to an OD_600_ of 0.1 in 50 ml LoFlo_glu_ medium and grown at 30°C for 8 h. For the wild-type SC5314 strain, BPS (0.05 mg/ml) and ferrozine (5 mM) were also added to the medium. Cells were harvested and washed with ice-cold water. After resuspension in TRIzol (ThermoFisher), cells were broken with glass beads using a FastPrep machine (MP Biomedicals). The addition of chloroform, isopropanol, and three washes with 70% ethanol resulted in the isolation of pure RNA. The RNA concentration was quantified using a NanoDrop spectrophotometer (ND-1000; Isogen Life Science). Equal amounts of RNA were treated with DNase (New England Biolabs) and then converted to cDNA using the iScript cDNA synthesis kit (Bio-Rad). Real-time quantitative PCRs (qRT-PCR) were performed using GoTaq polymerase (Promega) and the StepOnePlus real-time PCR device (ThermoFisher). Primers used for qPCR are listed in Table S3. qBasePlus software was used for gene expression analysis. Statistical analysis of the results was performed using GraphPad Prism. Expression data were log_2_(Y) transformed to enable the use of standard statistical methods. Graphs show the untransformed data with the standard errors of the means (SEM). The statistical method used is depicted under each graph.

### Mammalian cell culture.

HeLa cells, derived from a human cervical carcinoma, were kindly donated by Frank Claessens (KU Leuven). These cells were cultured in Dulbecco’s modified Eagle medium (DMEM) from Gibco, complemented with GlutaMAX (Gibco), 10% fetal bovine serum, and gentamicin (Gibco). Cells were seeded in cell culture flasks and incubated at 37°C and 5% CO_2_ in air.

### Mammalian cell toxicity determination.

Cell toxicity imposed by C. albicans cells was measured through release of LDH in the culture supernatant by using the CyQUANT LDH cytotoxicity assay from ThermoFisher. HeLa cells were seeded in special 96-well microtiter plates (Nunclon Delta Surface; ThermoFisher) at 10^5^ cells per well in 100 μl and allowed to adhere to the bottom for 24 h at 37°C and under 5% CO_2_. *Candida* cells were added to a final concentration of 10^5^ cells per well and allowed to infect the cells for another 24 h under the same conditions. According to the manufacturer’s guidelines, lysis buffer was added to a few wells as a positive control and phosphate-buffered saline (PBS) as a negative control. After incubation for 45 min, 50 μl of the cell supernatant was transferred to a normal 96-well microtiter plate, and 50 μl of reaction mixture was added. After 30 min of incubation at room temperature and protected from light, 50 μl stop buffer was added and the OD was determined spectrophotometrically at 490 and 680 nm. The toxicity in wells where lysis buffer was added was set to 100%, and the toxicity imposed by the *Candida* cells was determined relative to this control.

### Adhesion to mammalian cells.

Adhesion of *Candida* cells to mammalian HeLa cells was assessed using the following procedure. HeLa cells were seeded at a density of 10^5^ cells per well in 100 μl and allowed to adhere and grow for 48 h at 37°C and under 5% CO_2_. *Candida* cells were added to a final concentration of 10^5^ cells per well and allowed to infect the cells for 90 min under the same conditions. The cells were washed twice using PBS, and 25 μl trypsin was added per well and incubated for 10 min. After addition of 175 μl DMEM, cells were resuspended, diluted, and plated onto YPD agar plates. Numbers of CFU were determined.

### Mouse model of systemic infection.

All experiments involving mice were approved by the Ethical Committee for Animal Experimentation of KU Leuven. Each treatment group contained eight female BALB/c mice of 6 to 8 weeks of age. The sample size was determined based on analysis by G*Power software (47). The *Candida* strains were grown overnight in YPD, washed twice using PBS, and injected at 8.5 × 10^5^ cells per mouse via the tail vein. After infection, the mice were inspected daily for morbidity and mortality. Upon signs of severe morbidity, such as over 15% weight loss, severely hunched back, or ruffled fur, the mice were euthanized using isoflurane gas and cervical dislocation.

### Statistical analysis.

The sample size of the *in vitro* experiments varied from 3 to 4 samples within each group. For the SC5314 wild-type strain, individual colonies were taken as replicates. For mutant strains, independent transformants were isolated and used. Displayed parameters, statistical methods used, and corrections applied are clarified in the figure legends.
