# Supplementary figures and images for: Molecular Elucidation of Riboflavin Production and Regulation in Candida albicans, toward a Novel Antifungal Drug Target
Source: mSphere. 2020 Aug 5;5(4):e00714-20. doi: 10.1128/mSphere.00714-20 (PMC7407072; doi:10.1128/mSphere.00714-20)

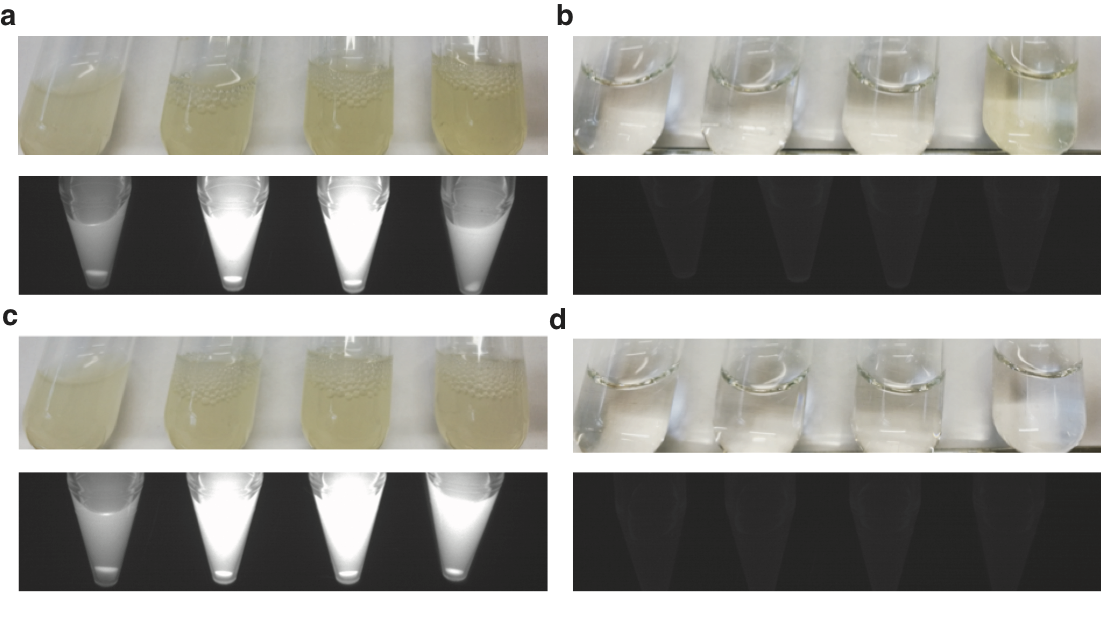

Supplement: FIG S4 [file mSphere.00714-20-sf004.tif]
