# Supplementary material for: Molecular Elucidation of Riboflavin Production and Regulation in Candida albicans, toward a Novel Antifungal Drug Target
Source: mSphere. 2020 Aug 5;5(4):e00714-20. doi: 10.1128/mSphere.00714-20 (PMC7407072; doi:10.1128/mSphere.00714-20)
Supplement: TABLE S1 [file mSphere.00714-20-st001.docx]

| **Strain name** | **Genotype** | **Reference** |
| --- | --- | --- |
| SC5314 | Clinical isolate *Candida albicans*, wild-type | (1) |
| *RIB1* ox | Same as SC5314, with 2 copies of CIp10-*CaRIB1-NAT1* integrated | This study |
| *RIB2* ox | Same as SC5314, with 2 to 3 copies of CIp10-*CaRIB2-NAT1* integrated | This study |
| *RIB3* ox | Same as SC5314, with 3 copies of CIp10-*CaRIB3-NAT1* integrated | This study |
| *RIB4* ox | Same as SC5314, with 4 to 6 copies of CIp10-*CaRIB4-NAT1* integrated | This study |
| *RIB5* ox | Same as SC5314, with 2 copies of CIp10-*CaRIB5-NAT1* integrated | This study |
| *RIB7* ox | Same as SC5314, with 2 copies of CIp10-*CaRIB7-NAT1* integrated | This study |
| *FMN1* ox | Same as SC5314, with 2 to 3 copies of CIp10-*CaFMN1-NAT1* integrated | This study |
| *TPK1* ox | Same as SC5314, with CIp10-*CaTPK1-NAT1* integrated | This study |
| *TPK2* ox | Same as SC5314, with CIp10-*CaTPK2-NAT1* integrated | This study |
| EV | Same as SC5314, with CIp10-*NAT1* integrated | This study |
| *Carib1*∆/∆ | Same as SC5314, with *Carib1*∆/∆ | This study |
| *Carib1*∆/∆ + *CaRIB1* | Same as *Carib1*∆/∆, with CIp10-*CaRIB1-NAT1* integrated | This study |
| *CaSEF1^S132A.S676A^* | Same as SC5314, with S132 and S676 replaced by alanine (TCA to GCC) | This study |
| S288c | Prototrophic *MAT****a****/MATα* strain | (2) |
| *Scrib1*∆ | Same as S288c, with *Scrib1∆/∆* | This study |
| *Scrib1*∆ + *CaRIB1* | Same as S288c, with Sc*RIB1/ScRIB1::CaRIB1/CaRIB1* | This study |
| *Casef1*∆/∆ | Same as SC5314, with *Casef1*∆/∆ | This study |
| *Casef1*∆/∆ + *CaSEF1* | Same as *Casef1*∆/∆, with CIp10-*CaSEF1-NAT1* integrated | This study |

1. Fonzi WA, Irwin MY. 1993. Isogenic strain construction and gene mapping in *Candida albicans*. Genetics 134:717-28.

2. Engel SR, Dietrich FS, Fisk DG, Binkley G, Balakrishnan R, Costanzo MC, Dwight SS, Hitz BC, Karra K, Nash RS, Weng S, Wong ED, Lloyd P, Skrzypek MS, Miyasato SR, Simison M, Cherry JM. 2014. The reference genome sequence of *Saccharomyces cerevisiae*: then and now. G3 (Bethesda) 4:389-98.
