# Supplementary material for: Molecular Elucidation of Riboflavin Production and Regulation in Candida albicans, toward a Novel Antifungal Drug Target
Source: mSphere. 2020 Aug 5;5(4):e00714-20. doi: 10.1128/mSphere.00714-20 (PMC7407072; doi:10.1128/mSphere.00714-20)
Supplement: TABLE S2 [file mSphere.00714-20-st002.docx]

| **Overexpression plasmids** | | | | |
| --- | --- | --- | --- | --- |
| **Plasmid name** | **Backbone** | **Insert** | **Marker** | **Reference** |
| EV | CIp10-*NAT1* | / | *CaNAT1* - *AmpR* | (1) |
| CIp10-*CaRIB1-NAT1* | CIp10-*NAT1* | *CaRIB1* | *CaNAT1* - *AmpR* | This study |
| CIp10-*CaRIB2-NAT1* | CIp10-*NAT1* | *CaRIB2* | *CaNAT1* - *AmpR* | This study |
| CIp10-*CaRIB3-NAT1* | CIp10-*NAT1* | *CaRIB3* | *CaNAT1* - *AmpR* | This study |
| CIp10-*CaRIB4-NAT1* | CIp10-*NAT1* | *CaRIB4* | *CaNAT1* - *AmpR* | This study |
| CIp10-*CaRIB5-NAT1* | CIp10-*NAT1* | *CaRIB5* | *CaNAT1* - *AmpR* | This study |
| CIp10-*CaRIB7-NAT1* | CIp10-*NAT1* | *CaRIB7* | *CaNAT1* - *AmpR* | This study |
| CIp10-*CaFMN1-NAT1* | CIp10-*NAT1* | *CaFMN1* | *CaNAT1* - *AmpR* | This study |
| CIp10-*CaTPK1-NAT1* | CIp10-*NAT1* | *CaTPK1* | *CaNAT1* - *AmpR* | This study |
| CIp10-*CaTPK2-NAT1* | CIp10-*NAT1* | *CaTPK2* | *CaNAT1* - *AmpR* | This study |
| CIp10-*CaSEF1-NAT1* | CIp10-*NAT1* | *CaSEF1* | *CaNAT1* - *AmpR* | This study |
| **CRISPR-Cas9 plasmids** | | | | |
| **Plasmid name** | **Description** | | **Marker** | **Reference** |
| pADH99 | *Ca* HIS/FLP *CAS9* expression | | *AmpR* | (2) |
| pADH110 | *Ca* template fragment A | | *AmpR* | (2) |
| pADH147 | *Ca* template fragment B | | *AmpR* | (2) |
| p*TEF*-*CAS9*-*BLE* | *Sc CAS9* expression | | *BLE* - *AmpR* | (3) |
| pgRNA-uni-*KanMX* | *Sc* gRNA plasmid | | *KanMX* - *AmpR* | (3) |
| pgRNA-G1-*KanMX* | *Sc* gRNA plasmid with G1 site | | *KanMX* - *AmpR* | This study |
| pTOPO-G1-*NatMX*-G1 | *Sc* template G1-*NatMX*-G1 | | *NatMX* - *AmpR* | J. Thevelein |
| pTOPO-G1-*HPH*-G1 | *Sc* template G1-*HPH*-G1 | | *HPH* - *AmpR* | J. Thevelein |

1. Demuyser L, Swinnen E, Fiori A, Herrera-Malaver B, Vestrepen K, Van Dijck P. 2017. Mitochondrial Cochaperone Mge1 Is Involved in Regulating Susceptibility to Fluconazole in *Saccharomyces cerevisiae* and *Candida* Species. MBio 8.

2. Nguyen N, Quail MMF, Hernday AD. 2017. An Efficient, Rapid, and Recyclable System for CRISPR-Mediated Genome Editing in *Candida albicans*. mSphere 2.

3. Cen Y, Timmermans B, Souffriau B, Thevelein JM, Van Dijck P. 2017. Comparison of genome engineering using the CRISPR-Cas9 system in *C. glabrata* wild-type and *lig4* strains. Fungal Genet Biol 107:44-50.
