# Supplementary material for: Molecular Elucidation of Riboflavin Production and Regulation in Candida albicans, toward a Novel Antifungal Drug Target
Source: mSphere. 2020 Aug 5;5(4):e00714-20. doi: 10.1128/mSphere.00714-20 (PMC7407072; doi:10.1128/mSphere.00714-20)
Supplement: TABLE S3 [file mSphere.00714-20-st003.docx]

| **Primer name** | **Sequence 5’ -> 3’** |
| --- | --- |
| **Primers used for generating CIp10 overexpression plasmids** | |
| *CaTPK2*_Fw_NheI | CTAGCTAGCGCAATGGTGAATCTTTTAAAGAAAC |
| *CaTPK2*_Rev_AatII | CCCGACGTCGGGAAAGTCAAGGAAATACAGAGC |
| *CaTPK1*_Fw_NheI | CTAGCTAGCGCAATGACATCCATGGAACCAGCAG |
| *CaTPK1*_Rev_ClaI  *CaRIB1*_Fw_NheI  *CaRIB1*_Rev_AatII  *CaRIB2*_Fw_NheI  *CaRIB2*_Rev_ AatII  *CaRIB3*_Fw_NheI  *CaRIB3*_Rev_ AatII  *CaRIB4*_Fw_NheI  *CaRIB4*_Rev_ AatII  *CaRIB5*_Fw_NheI  *CaRIB5*_Rev_ AatII  *CaRIB7*_Fw_NheI  *CaRIB7*_Rev_ AatII  *CaFMN1*_Fw_NheI  *CaFMN1*_Rev_ AatII  *CaSEF1*_Fw_NheI  *CaSEF1*_Rev_ AatII | CCATCGATGGAATTAAAAGTCCTGGAATTGATCAC  CATTCAARAATGCTGCAGGCTAGCATGACATCGATAAAACATCCAA  CGAGGTCGACGGTATCGATGACGTCTTAGATTTTGATGGGTTTCTCTA  CATTCAAAATGCTGCAGGCTAGCATGTCAATATACAAGAGTGGCT  CGAGGTCGACGGTATCGATGACGTCTTATTCTTGAATATGTTCATGACC  CATTCAAAATGCTGCAGGCTAGCATGATCAGTATGACTAACATCTTTA  CGAGGTCGACGGTATCGATGACGTCTTATTTAGAAATATATTCAACTAATTG  CATTCAAAATGCTGCAGGCTAGCATGACCAAAACTGATAATCAGG  CGAGGTCGACGGTATCGATGACGTCTTAATTGAATTTAGTGGCCATTT  CATTCAAAATGCTGCAGGCTAGCATGTTTACTGGTCTTGTTGAAAC  CGAGGTCGACGGTATCGATGACGTCTTACTTGATATATTCTTTAACTTTCT  CATTCAAAATGCTGCAGGCTAGCATGTCATTAATCCCATTACCTG  CGAGGTCGACGGTATCGATGACGTCCTATTGATGATAATCAATTCTAG  CATTCAAAATGCTGCAGGCTAGCATGACAGTTTCTAACAACACTT  CGAGGTCGACGGTATCGATGACGTCTCATTTCGTGAAATATTCCTTGC  CATTCAAAATGCTGCAGGCTAGCATGAAGTTTGAAAAAGGTAAAGTG  CGAGGTCGACGGTATCGATGACGTCTTATTTCTCTTGCATCATATTAAC |
| **Primers used for copy number determination via qPCR** | |
| *CaACT1*pr_Fw | GGCTATGCCAATCAAAAAGG |
| *CaACT1*pr_Rev | CCCCTTGGCCATAGGATATT |
| *CaACT1*_Ref_Fw | TGTTGGTGATGAAGCCCAATC |
| *CaACT1*_Ref_Rev | CATATCGTCCCAGTTGGAAACAA |
| *Ca18S*_Ref_Fw | GATGCCCTTAGACGTTCTGG |
| *Ca18S*_Ref_Rev | CACGACGGAGTTTCACAAGA |
| *CaTEF1*_Ref_Fw | CCACTGAAGTCAAGTCCGTTGA |
| *CaTEF1*_Ref_Rev | CACCTTCAGCCAATTGTTCGT |
| **Primers used for qRT-PCR** | |
| *CaRIB1*_Fw | GTGCCGATACAGTGGAAGC |
| *CaRIB1*_Rev | CCCAAATCAACCAAGATAGCC |
| *CaRIB2*_Fw | AGGTAATATCGACGGGTCATTC |
| *CaRIB2*_Rev | GGTACTTCTCTTTCCCCACCA |
| *CaRIB3*_Fw | TACCGTTAAGAGCCGTTCCA |
| *CaRIB3*_Rev | CAAATCACCCCAGCAGGTT |
| *CaRIB4*_Fw | GGCATTCCTGTTATTTTTGGTG |
| *CaRIB4*_Rev | TGTGCATTTTGCCTTCAATC |
| *CaRIB5*_Fw | TCGACAAGAAACCAGATGGA |
| *CaRIB5*_Rev | ATGAAGCCCCATCAATAGCA |
| *CaRIB7*_Fw | TTGGGACCATTTTAGCTGATG |
| *CaRIB7*_Rev | TGGTCTGATTGATGCGAATA |
| *CaFMN1*_Fw | TCTGAACTAGGAATCCCCACA |
| *CaFMN1*_Rev | TTCGTCACATTGGGCTGTTA |
| *CaSEF1*_Fw | GGGCCAATGAGTTACACGAC |
| *CaSEF1*_Rev | TGATACAATTCGGTGGCAGA |
| *CaACT1*_Ref_Fw | TGTTGGTGATGAAGCCCAATC |
| *CaACT1*_Ref_Rev | CATATCGTCCCAGTTGGAAACAA |
| *Ca18S*_Ref_Fw | GATGCCCTTAGACGTTCTGG |
| *Ca18S*_Ref_Rev | CACGACGGAGTTTCACAAGA |
| *CaEFB1*_Ref_Fw | GCTGCCAAATCTATTGTCACCT |
| *CaEFB1*_Ref_Rev | TGAGCACCCCAAGTCAAAC |
| *CaLSC2*_Ref_Fw | CTGCCACCAAGAACTTCAAC |
| *CaLSC2*_Ref_Rev | CAGCTGGGTCCAAATCTTCG |
| *CaSAP2*_Ref_Fw | TGTAATTTGTCAGGGGATGTTG |
| *CaSAP2*_Ref_Rev | CACCTTGTAAAGAAGCAGCAAA |
| *CaTDH3*_Ref_Fw | ATCCCACAAGGACTGGAGAG |
| *CaTDH3*_Ref_Rev | CCTTACCAACGGCTTTAGCA |
| *CaTUB1_*Ref_Fw | TTACCCAGCTCCACAAGTGTC |
| *CaTUB1*_Ref_Rev | AAGTACAATCGGCGTGTTCC |
| **Primers used for making *C. albicans* mutant strains** | |
| gRNA_*RIB1*  gRNA_*SEF1*  gRNA_mut S132_*SEF1*  gRNA_mut S676_*SEF1*  donor Fw_*RIB1*_del  donor Rev_*RIB1*_del  donor Fw_*SEF1*_del  donor Rev_*SEF1*_del  donor Fw_*SEF1*_S132A  donor Rev_*SEF1*_S132A  donor Fw_*SEF1*_S676A  donor Rev_*SEF*_S676A | CGTAAACTATTTTTAATTTGTACATTGGTAGATTGACACCGTTTTAGAGCTAGAAATAG  CGTAAACTATTTTTAATTTGTTACAATTAGGTCTACATCGGTTTTAGAGCTAGAAATAG  CGTAAACTATTTTTAATTTGTGGATTTGTGACCCTTTGCGGTTTTAGAGCTAGAAATAG  CGTAAACTATTTTTAATTTGAAGACGATCAGTACTTGATAGTTTTAGAGCTAGAAATAG  AATCTACCACAACCCATTTAATAAATTTAATTGCGCTGATTCAAATTCAAACTAAAGAAAAAAAAACATTGTTATGGAGTTGATGAGAT  ATCTCATCAACTCCATAACAATGTTTTTTTTTCTTTAGTTTGAATTTGAATCAGCGCAATTAAATTTATTAAATGGGTTGTGGTAGATT  CTTAACTAGAACCATAGAACCTCAACATTTGTTTCTATAGAAAAGATTCTACCTCATTAGAATGTAAATAGTTAGTAATTATGTTAATA  TATTAACATAATTACTAACTATTTACATTCTAATGAGGTAGAATCTTTTCTATAGAAACAAATGTTGAGGTTCTATGGTTCTAGTTAAG  GTTTGAAATGTGAAATTGACCCCGAATTTAGACCTCGCAAAGGGGCCCAAATCCAATCATTGAAACTGGATGTTGATGAATTGAAGGCC  GGCCTTCAATTCATCAACATCCAGTTTCAATGATTGGATTTGGGCCCCTTTGCGAGGTCTAAATTCGGGGTCAATTTCACATTTCAAAC  TCTATGATTTGGTTTGGTGTGTACATGAGGCAAGAAGACGAGCCGTACTTGATAAGGGGAAAAGACAGGCTCAGCCTAACAAGAAAATC  GATTTTCTTGTTAGGCTGAGCCTGTCTTTTCCCCTTATCAAGTACGGCTCGTCTTCTTGCCTCATGTACACACCAAACCAAATCATAGA |
| **Primers used for making *S. cerevisiae* mutant strains** | |
| *ScRIB1*_Fw_del  *ScRIB1*_Rev_del  donor Fw_*ScRIB1*_del  donor Rev_ *ScRIB1*_del  *CaRIB1*_Fw_integration  *CaRIB1*_Rev_integration  G1-cloning | TGGACAATAATTGAGTCATTACAAAAAGTGGCGTACATAAAACTACAACAAACCTACAGGGTGGTCGGCTGGAGATCGG  TATTTTCGTTTCATTACAAAAAAAGCACTATATGTACTAATAATTATGCTACACTTGTGTAGCCGTTATGGCGGGCATC  CATTACAAAAAGTGGCGTACATAAAACTACAACAAACCTACAGGACACAAGTGTAGCATAATTATTAGTACATATAGTGCTTTTTTTGT  ACAAAAAAAGCACTATATGTACTAATAATTATGCTACACTTGTGTCCTGTAGGTTTGTTGTAGTTTTATGTACGCCACTTTTTGTAATG  TGGACAATAATTGAGTCATTACAAAAAGTGGCGTACATAAAACTACAACAAACCTACAGGATGACATCGATAAAACATCCAA  TATTTTCGTTTCATTACAAAAAAAGCACTATATGTACTAATAATTATGCTACACTTGTGTTTAGATTTTGATGGGTTTCTCTA  GCAGTGAAAGATAAATGATCGGCTGATTTTCGCAGTTCGGGTTTTAGAGCTAGAAATAG |
